# Evidence of pre-existing active Zika virus circulation in Sudan prior to 2012

**DOI:** 10.1186/s13104-018-4027-9

**Published:** 2018-12-19

**Authors:** Mohammed A. Soghaier, Deena M. Abdelgadir, Sozan M. Abdelkhalig, Hamoda Kafi, Isam M. A. Zarroug, Amadou A. Sall, Mawahib H. Eldegai, Rehab M. Elageb, Muntasir M. Osman, Hayat Khogali

**Affiliations:** 1 Epidemiology and Zoonotic Diseases Department, Federal Ministry of Health, Osman Digna Street with Nile Avenue, PO Box 303, 1111 Khartoum, Sudan; 2 The Department of Epidemiology, National Public Health Laboratory, Federal Ministry of Heath, Khartoum, Sudan; 3 Department of Integrated Vector Management, Federal Ministry of Health, Khartoum, Sudan; 4 The Department of Medical Entomology, National Public Health Laboratory, Federal Ministry of Health, Khartoum, Sudan; 5 Institut Pasteur de Dakar, Dakar, Senegal

**Keywords:** Zika IgG, Yellow fever, Arboviruses, Aedes mosquito

## Abstract

**Objective:**

The purpose of this study is to provide the first evidence of Zika virus circulation (ZIK) in Sudan. Zika virus was first isolated in the Zika forest of Uganda in 1947, and in 2016, the World Health Assembly declared it a public health emergency of international concern. The discovery of Zika virus circulation in Sudan came as a secondary finding in a 2012 country-wide yellow fever prevalence study, when laboratory tests were done to exclude cross-reactions between flaviviruses. The study was cross-sectional community-based, with randomly selected participants through multi-stage cluster sampling. A sub-set of samples were tested for the Zika virus using ELISA, and the ones that demonstrated reactive results were subsequently tested by PRNT.

**Results:**

The prevalence of Zika IgG antibodies among ELISA-tested samples was 62.7% (59.4 to 66.1, 95% CI), and only one sample was found positive when tested by PRNT. This provided the first documented evidence for the pre-existing circulation of Zika virus circulation in Sudan. This evidence provides the foundation for future research in this field, and further structured studies should be conducted to determine the epidemiology and burden of the disease.

## Introduction

Zika virus (ZIK) is a positive-sense RNA arbovirus belongs to the Flaviviridae family of over 70 viruses, including yellow fever (YF), dengue fever (DF), and West Nile (WN) virus [1–3]. ZIK is an arbovirus, transmitted by females of the of the Aedes genus mosquitos, including *A. aegypti* and *A. albopictus* which are also known to be responsible for the transmission of YF and DF. There is suggestive evidence for ZIK to be transmitted through blood, urine, or sexual contact [4–6].

ZIK was first isolated in the Zika forest of Uganda from Rhesus monkeys in 1947 and the Aedes mosquito in 1948 [7, 8]. The first human ZIK infection was reported in 1954, in Nigeria [8, 9]. The disease spread from Uganda to western Africa and Asia in the middle of the 20th century before reaching other regions where the cases were mild, and no deaths or hospitalizations were reported [10, 11]. In 2016, however, the disease was declared as a public health emergency of international concern (PHEIC) due to its rapid spread and catastrophic implications [12–14]. Epidemiologists established a causal relationship between the virus and incident cases of microcephaly [15, 16].

ZIK was previously detected in Egypt, Cameroon, and several countries in Asia [17–21], however no study had been conducted in Sudan to identify the circulation of the virus despite the country having comprehensive knowledge and estimates regarding the circulation of other flaviviruses. Sudan has a history of major outbreaks of YF and DF that bring massive reported mortalities and significant economic burden [22, 23], and has identified the Aedes mosquito in all parts of the country since 1908 [24]. The Aedes mosquito in Sudan has demonstrated high adaptability and efficiency in transmitting flaviviruses [25, 26].

In 2012, a community-based serological study was conducted in Sudan, aiming to assess and estimate the risk of YF following a major YF outbreak in the Darfur region that same year. When laboratory tests were conducted to exclude cross-reactions between flaviviruses, the study confirmed the presence, and active circulation, of ZIK as a secondary outcome. This report aims to provide evidence of ZIK circulation in Sudan.

## Main text

### Methods

#### Study design

It was a cross-sectional community-based study conducted in 10 out of 17 administrative states of the country after excluding states recently covered by YF vaccination. Individuals aged below 9 months were included, while visitors from other provinces of Sudan who were recently targeted by YF mass vaccination, patients with chronic debilitating diseases such as cancers, and individuals taking immunosuppressant medications were excluded.

#### Sampling technique

A multi-stage cluster sampling technique was employed in this study [27]. First, four distinct ecological zones were identified in the country based on rainfall, vegetation, and altitude in order to account for differences in humidity, temperature, and land-cover. Second, a random point generator in Arc GIS (Geographic Information System) was run, and it randomly selected two locations per zone to sample [28]. Using the geocoordinates of each randomly selected point, the towns and villages closest to that point were identified using Google Earth. Third, a random number generator table was used to select households, and within each household, one participant was selected from the eligible population using a simple random sampling approach (random generating table).

#### Sample size

The sample size was calculated per ecological zone based on the population and the estimated seroprevalence of YF antibodies in that particular region. Approximately 30% oversampling was done to compensate for the households that were unavailable for sampling and 15% for individual refusal. A design effect of 2 was used to account for clustering, and the formula for the calculation of sample size was applied using the OpenEpi software [29]. A total of 1775 participants were interviewed and sampled to estimate the prevalence of YF, and all positive samples were subsequently tested for other flaviviruses to exclude cross-ration.

#### Data collection and statistical analysis

A structured questionnaire was used to collect information regarding socio-demographic variables. Venous blood samples were collected for viral serotyping. Descriptive and inferential statistics were conducted; a multivariate logistic regression model was run using STATA-12 software. The strength of association between Zika IgG and important risk factors is reported in the form of odds ratio (OR) and 95% confidence interval.

#### Laboratory analysis and outcome determination

Serum specimens were tested for YFV-specific IgG/IgM antibodies using enzyme-linked immunoassay (ELISA). Given the potential for cross-reactivity within the flaviviruses, YFV positive samples were assessed by ELISA for antibodies against ZIK, DF, and WN virus. Samples that tested positive for DF viruses or ZIK were then tested for neutralizing antibodies using plaque reduction; neutralization testing (PRNT). A total of 845 samples out of 861 YFV-positive samples were subsequently tested for Zika IgG using ELISA and PRNT. The tests were conducted at the Institut Pasteur de Dakar, Senegal for Zika and other differential diagnoses of flaviviruses using specific in-house ELISA method [30, 31]. Positive and negative control sera were prepared, and 96 well plates were coated with mouse hyperimmune ascitic fluids, incubated at 4 °C overnight, and then washed with PBS and 0.05% Tween. Specific ZIK antigens were added, and the plates were incubated for 1 h at 37 °C. Serum samples and controls were then added to the wells, and the plates were incubated for 1 h at 37 °C. After washing, the anti-human IgG antibodies conjugated with horseradish peroxidase were added. The specific substrate was added as well, and the reaction was stopped with sulphuric acid. An ELISA microplate reader was used to measure the Optical Density (OD) and identify the positive samples. For PRNT, the serum samples were deactivated through gentle heating for 30 min and diluted at 1:10 to 1:640. Screening for neutralizing antibodies was performed using ZiKV ref. MR766 and YFV ref. 17D. For both viruses, a stock of 1000 Plaque Forming Units (PFU)/ml was prepared. Sera were mixed with an equal volume of the viral stock and added to the plates. Susceptible cells were then added to the wells and incubated at 37 °C. The plates were overlaid with methylcellulose and incubated for 5 days at 37 °C. Following this, a second overlay, containing 0.5% neutral red, was added to visualize and count plaques, and 90% PRNT titers were calculated as the reciprocal of the maximum serum dilution capable of neutralizing 90% or more of the tested virus [32].

### Results

Out of 1775 participants, 97% were found to have sufficient samples and agreed to being tested. Overall, 47% of participants were males, and the mean age of the participants was 36.9 years, ranging from 2 to 92 years. The baseline characteristics of participants are summarized in Table 1. Out of the tested samples, 861 were reactive to YF and 845 were subsequently tested for other flaviviruses, including ZIK. In descriptive statistics, out of the 845 samples tested for Zika IgG, 530 or 62.7% were found positive (59.4 to 66.1, 95% CI). The occurrence of Zika IgG positive samples was slightly higher in female participants (67% females and 59% males). The older age groups (above 40 years) were found to have a higher proportion of positive samples (65%) as compared to younger age groups. Table 2 provides details of the descriptive statistics. Only one sample tested positive for Zika by PRNT; he was a 50-year-old male from Shendi city, one of the largest cities within the Nile River state.

**Table 1:** Baseline characteristics of study participants and distribution by ecological zones

	Zone 1	Zone 2	Zone 3	Zone 4	Total
Participants	n = 654 (%)	n = 656 (%)	n = 181 (%)	n = 284 (%)	n = 1775(%)
Urban site 1	Abri: 252 (39)	Port Sudan: 292 (45)	Omdurman: 23 (13)	Kosti: 106 (37)	Urban: 1496 (84) Rural: 279 (16)
Rural site 1	Amara: 79 (12)	Hoshiri: 39 (6)	Elsalamania: 43 (24)	Umahani: 15 (5)	
Urban site 2	Barbar: 300 (46)	Tokkar: 309 (47)	Shendi: 85 (47)	Adamazin: 129 (46)	
Rural site 2	Elferakha: 23 (3)	Ashad: 16 (2)	Dimelgarri: 30 (16)	Agadi: 34 (12)	
Sex					
Male	247 (38)	354 (54)	84 (46)	142 (50)	826 (47)
Female	407 (62)	302 (46)	97 (54)	142 (50)	949 (53)
Age group (years)					
< 15	41 (6)	33 (5)	18 (10)	80 (28)	172 (10)
15–39	361 (55)	323 (49)	90 (50)	133 (47)	907 (51)
40–65	243 (37)	259 (40)	61 (34)	61 (23)	656 (53)
> 65	9 (2)	41 (6)	12 (6)	3 (2)	6 (4)

**Table 2:** Distribution of Zika virus ELISA IgG positive samples by ecological zones, residential sites, and demographical factors

Zone	Village	Total tested	Positive	Proportion of positive (%)
Zone 1	Abri^a^	58	42	72
	Amara	16	1	6
	Barbar^a^	106	60	57
	Elferaikha	11	9	82
	Subtotal	191	112	59
Zone 2	Port Sudan^a^	206	124	60
	Hoshiri	12	10	83
	Tokkar^a^	210	148	70
	Ashad	16	7	44
	Subtotal	444	289	65
Zone 3	Omdurman^a^	8	4	50
	Elsalamania	16	8	50
	Shendi^a^	24	18	75
	Dimelgaraia	19	12	63
	Subtotal	67	42	63
Zone 4	Kosti^a^	25	17	68
	Umahani	9	2	22
	Adamazin^a^	78	44	56
	Agadi	31	24	77
	Subtotal	143	87	61
Age (years)	< 15	64	30	47
	15–39	417	265	64
	40–65	327	211	65
	> 65	37	24	65
Sex	Males	432	254	59
	Females	413	276	67
Grand total		845	530	63

^a^Urban settlements

With regard to inferential statistics, age was found to be the most important variable in predicting ZIK with OR 2.1 (1.2–3.7, 95% CI) for the age group 15–39 years and OR 2.2 (1.2–3.9, 95% CI) for the age group 40–65 years in comparison with the youngest age group, which was below 15 years. The male sex and urban residence were correlated to ZIK with a slight difference from females and residents of rural settlements. There is no significant difference in the proportion of Zika IgG antibodies across the geo-ecological zones identified in this study. Table 3 summarises the multivariate logistic regression model testing the association between Zika and key factors.

**Table 3:** Summary for the multivariate logistic regression model testing association between Zika IgG and key factors

Factors	Odds ratio	Z-statistic	P-value*	95% confidence interval
Geographical zone				
Zone (2)	1.2	1.01	0.31	0.84–1.71
Zone (3)	1.3	0.91	0.36	0.72–2.42
Zone (4)	1.4	1.29	0.19	0.85–2.17
Urban/rural residence				
Urban	1.4	1.66	0.09	0.93–2.14
Age group (years)				
15–39	2.1	2.61	0.01	1.20–3.70
40–65	2.2	2.63	0.01	1.21–3.86
> 65	2.2	1.76	0.07	0.92–5.14
Sex				
Male	1.3	1.84	0.06	0.98–1.74

Reference groups are: Zone (1), rural settlements, younger age group < 15 years and female sex

* P-value cut-off for statistical significance ≤  0.05

### Discussion

These findings reveal the first evidence of active ZIK transmission in Sudan. The age of the participants is the principal factor predicting ZIK in this study, indicating that the risk of ZIK increases with age. The participants in age groups older than 15 years had at least a two times higher risk for contracting ZIK as compared to participants in the age group below 15 years. This finding is coherent with the epidemiology of vector-borne diseases, since cumulative exposure increases with age. This finding is confirmed by the findings of other studies as well [33]. Factors such as setting and sex did not significantly predict ZIK. Some other studies have found females to be more affected by the disease, and urban settings to be related to ZIK due to vector ecology [4, 33]. There is no difference in association between ZIK and ecological zones. It was assumed that the zone (2) in the eastern part of the country near the Red Sea coast would have a higher proportion of ZIK, since this zone is known to be the region witnessing the highest density and activity of the *A. aegypti* mosquito, the vector responsible for the transmission of a majority of arboviruses [22, 26].

The only PRNT-positive ZIK case detected in this study was a 50-year-old male from Shendi city, one of the largest cities within the Nile River state. The participant did not report any history of travel or illness during the 6 months prior to the study. Shendi is located on the eastern bank of the Nile River, approximately 100 miles northeast of Khartoum [34]. Shendi has no reported history of arboviral outbreaks, but neighbouring provinces, such as Merawi and Karima, witnessed a YF outbreak in 1989 and confirmed cases of Rift Valley fever during the 2007 outbreak [35–37]. The Nile River state has excessive population movement with all the other parts of the country, and recently witnessed a huge influx of workers attracted by traditional gold mining activities [38].

Sudan is known to have several arboviruses circulating across the country. YF has been documented in Sudan since the 1940s with evidence of repeated outbreaks—some that were extremely catastrophic, such as the 2012 outbreak [23]. DF and DHF were documented to be actively circulating across the country, with major outbreaks in the eastern parts near the Red Sea and Kassala states [26, 39]. Active DF circulation was also documented in the western and southern parts along the new border of the Republic of South Sudan [40]. WN, Rift Valley (RV), and Chikungunya viruses were documented in Sudan as well, with major outbreaks such as the 2007 Rift Valley Fever (RVF) outbreak [41].

The presence of an arbovirus is a highly suggestive factor for circulation of other viruses due to common transmitting vector. In Sudan, the Aedes mosquito is present in all parts of the country with extremely active breeding near the Red Sea and Kassala states [22]. It was also detected in the central part of the country, including the capital city of Khartoum [25]. Between 2008 and 2010, the Red Sea state witnessed major incidents including increased morbidities among pregnant women, abortions, and stillbirths. The outbreak was determined to be a concomitant outbreak of DF in conjunction with Hepatitis E [42–44], however, samples were not tested for ZIK. Considering cross-reactivity, the question that arises is whether there was a correlation between the incidents during that 2-year period and ZIK? This would be one of the recommendations of the current discussion, to trigger thought and encourage further research in this field.

### Conclusion

This study established evidence of pre-existing active ZIK circulation in Sudan in 2012 and lays the foundation for future research in this field in the country.

### Limitations

Despite the originality and the important evidence disseminated by this publication, the reported evidence of ZIK circulation in Sudan was obtained as a secondary finding of a study that was primarily designed to estimate the risk of YF in the country. Only a subset of the overall participants was tested for ZIK, therefore the study lacks the power and generalizability to estimate the prevalence of the disease.

## Abbreviations

CI: confidence interval
DF: dengue fever
DHF: dengue haemorrhagic fever
ELISA: enzyme-linked immunoassay
GIS: geographic information system
IgG: immunoglobulin G
IgM: immunoglobulin M
OR: odds ratio
PHEIC: public health emergency of international concern
PRNT: plaque reduction neutralization testing
WN: west Nile
YF: yellow fever
ZIK: Zika virus
